# Clinical Studies with Large Non-Emetic Doses of Ipecacuanha: With a Contribution to the Therapeusis of Cholera

**Published:** 1875-02

**Authors:** Alfred A. Woodhull

**Affiliations:** Assistant Surgeon U. S. Army


### CLINICAL STUDIES WITH LARGE NON-EMETIC DOSES OF IPECACUANHA: With a Contribution to the Therapeusis of Cholera.

By ALFRED A. WOODHULL, M.D., Assistant Surgeon U. S. Army.

*A Special Report to the Surgeon General U. S. Army, read before the Atlanta Academy of Medicine.*

These clinical notes, and what seems to me a reasonable deduction from them and from certain appended quotations, are respectfully offered for the consideration of the profession.

Much that follows is not new, but merely corroborates the recorded experience of others. Reading and observation, however, teach me that the influence that ipecacuanha exerts over dysentery and certain forms of diarrhœa is not practically recognized by many physicians who, while they have read of its power, hesitate to employ the remedy. Notes of such cases may, therefore, be useful by inviting renewed attention to a subject which, in some parts of our country, is of vital interest. The opportunities at my disposal were taken advantage of to press the use of the drug further than is commonly done; and, that others may form an intelligent judgment, all the circumstances attending its administration have been carefully noted, although in some instances it may be that no positive benefit followed, and the cases have been narrated in greater detail than would be proper in a report simply covering admitted therapeutic ground.

It follows, therefore, that, besides the confirmatory cases, others are recorded which, so far as I know, belong to a new class. Whatever similar experience other medical men may have had, this is certainly original; and attention is invited to the route by which it was reached, but especially to the results attained. Finally, a suggestion of possible value is advanced for the consideration of those who may have the opportunity to use it. The cases are entered chronologically. All in which the drug was used in this way are reported, and no essential condition that was observed, either for or against the method, is omitted.

Having had at other posts a moderate experience that was favorable in the treatment of dysentery by large doses of ipecacuanha, and being acquainted with the East Indian reports on the subject, I resorted to it here at the beginning of the warm season.

Case I.—C. D., who had suffered with a severe diarrhœa several days, came upon the sick list 19th May, 1874, and was at once admitted to hospital. He had fever, severe pain in the bowels, small bloody discharges with straining—in short, dysentery. He was at once put to bed, and was given tinct. opii *m.* xv., followed in half an hour by pulv. ipecacuanhæ gr. xx., in as little water as possible, and a sinapism was applied to the stomach. This was repeated four hours afterward. There was no emesis; the bloody discharges at once ceased; the pain and tenesmus gradually passed away; a stool, soft but not dysenteric, occurred in the middle of the day; by evening he expressed himself as feeling very comfortable. No more medicine was given, but he was kept in bed two days. He was sent to quarters 22d, and returned to duty 24th May.

Case II.—J. H. F., a soldier of twenty years' service, seasoned, and in good general health, although an occasional hard drinker, came upon the sick report 29th May, 1874, with a typical case of acute dysentery. The attack was recent, but the symptoms were severe, and he was suffering extremely. He was immediately put to bed and was given tinct. opii *m.* xx., followed in twenty minutes by pulv. ipecac. gr. xxv., in a small quantity of water, and by a sinapism. The bloody stools ceased forthwith, and he began to perspire and to feel relieved. The dose was repeated in six hours, and ten grains more were given at 9 P. M. There was no vomiting. One or two copious evacuations, without pain and bloodless, occurred during the day, and by noon he declared himself much better and free from pain. The next day (30th), with no other treatment, he was quite well, but very weak. The prostration remained several days, but there was no return of the disease itself.

Case III.—A. H. was admitted to hospital 7th June, 1874, for a severe attack of diarrhœa with a dysenteric tendency. He was first given magnes. sulph. ℥iv., olei ricini f.℥ij., tr. opii *m.* v.; but no particular improvement following, the ipecacuanha was resorted to, as in the previous cases, with prompt relief. He was returned to quarters 10th, although a bronchial catarrh kept him on the report till 16th June.

Case IV.—W. H., in hospital with a severe diarrhœa. The ordinary remedies not relieving him, and as he complained of a severe pain in the transverse colon, although no blood was passed nor were there any direct symptoms of dysentery, the experiment was tried of giving, with the tincture of opium and mustard as usual, twenty-five grains of powdered ipecacuanha, at 9 P. M., 19th June, 1874. He slept well that night, felt much better the next day, was returned to quarters 21st, and to duty 23d June.

Case V.—B. R., admitted with a severe diarrhœa, 19th June, and, complaining almost identically with the preceding case, received two large doses of ipecacuanha, on 20th. One of these induced some vomiting, but the severe pain in the colon ceased, although the diarrhœa lingered a number of days longer. Returned to duty 9th July, 1874. (*See Case XV.*)

Case VI.—W. B. O. was admitted to hospital, 16th June, 1874, in a jaundiced condition from some functional hepatic derangement. He improved sufficiently to be returned to quarters 24th. At sick call, 27th, however, he was so changed in appearance and condition as to require immediate re-admission to hospital. He asserted that the greater part of the previous day and night he had suffered frequent and painful purgings and vomitings. He was at once (6:30 A.M.) given a bed. On visiting him at eight o'clock he said that he had had seven stools and as many attacks of vomiting within the past hour and a half. These were painful, the discharged matter was dark brown, there were constant abdominal pain and great thirst, his face was haggard and pinched, his skin was cold and bluish, his pulse very feeble, and his general condition one of great prostration.

Having Cases IV. and V. in mind, where severe abdominal pain, although much less in degree, subsided after the use of ipecacuanha, and believing, as I did, that this drug exerted a direct influence upon the intestinal excreting surface, and possibly upon the liver, I regarded this as a fair case in which to employ it, provided it could be retained. He was therefore at once given tr. opii *m.* xx. as preliminary. In a few minutes he threw up at least part of it, but pulv. ipecac. gr. xxv. was nevertheless administered, and a sinapism applied. This was retained, and the vomiting and purging at once ceased. By noon he felt remarkably better and was quite bright. One copious operation, without pain, occurred in the middle of the day. Pulv. ipecac. gr. xx. was repeated at 2 P.M., but, taking a cup of tea a couple of hours afterward, a part of it was then rejected. At night he said he "felt like a new man," and he certainly looked like a different person. There was no further trouble in this case from this acute attack; convalescence progressed regularly, and he was returned to duty in a few days.

CASE VII.—C. C. was admitted to hospital, with a well-marked case of ordinary acute dysentery, 1st July, 1874. The ipecacuanha treatment was at once adopted, and the bloody stools ceased with equal promptness. He remained in bed that day and got up on the 2d. On the 2d he had two stools before 4 P.M., each marked with a trace of blood. He was again put to bed and given, in the same manner, ipecacuanhæ gr. xx., which was easily retained, and the next day he was returned to duty.

It will be observed that, at first using this remedy after the familiar East Indian method, I was led to its employment in violent irritation of the colon without bloody discharges (Cases IV. and V.), and that in these it answered well. I was then induced to try it in the case (VI.) which resembled cholera morbus, from its manifestly good effects in violent intestinal irritation, and that here it was equally successful. By reflection upon these cases and upon its apparent mode of action, I formed the opinion that it might prove of service in cases of genuine cholera morbus, and also that it would be worthy a trial in Asiatic cholera. An early opportunity occurred to test it in the former disease.

CASE VIII.—E. B. was reported very sick, in his quarters, 9 A.M., 6th July, 1874. I found him lying on his bunk, complaining of great thirst and of severe cramps in his legs and feet, which men were rubbing. At this time his face was shrunken and bluish, his hands were blue and the ends of his fingers wrinkled, his skin was cold and clammy to the touch and was bathed in cold perspiration, his pulse was extremely feeble. I think that he might fairly be called in a state bordering upon collapse. He had fallen to the ground from the sudden and severe seizure of cramps in the legs a few minutes before. His Captain saw him while he was yet lying on the floor, and in describing him said: "His hands and face were blue, he appeared shrunken, he had severe cramps, and he looked to me like a corpse or a dying man." His general health had been good up to the preceding night; ate for dinner on 4th fresh mutton and blackberry pie; knows of no other exciting cause. About 10 P.M., 4th inst., was attacked with a painless diarrhœa, and between that hour and reveille he had eight or ten dejections, the first two or three of which were quite copious. He does not know their color. He had also four or five attacks of vomiting; does not know its appearance, as it was discharged out of doors in the dark. At sunrise he felt better, and did not think it necessary to report sick. Drank coffee for breakfast, threw it up, and the diarrhœa returned. Was intensely thirsty, but vomited all the water he drank. About 9 A.M., while walking to a water-pail, fell to the ground in a severe cramp. I then saw him, and had him immediately carried to the hospital, and withheld all fluids.

9:15 A.M.--Admitted hospital in the condition already described. Had epidemic cholera been prevalent, this would probably have been considered a case of that disease. The Hospital Steward, who had seen cholera asiatica, was at once reminded of it by his appearance. Temperature in the mouth 103 4-5° F.; pulse not counted, but very feeble. 9:30.—Given tr. opii *m.* xx., aquæ f.℥j. (The prescription was dispensed as written, but an error of the pen doubled the water.) A sinapism was put over the stomach. Eight or ten ounces of watery fluid was almost immediately vomited; nevertheless he took at 9:50 pulv. ipecac. gr. xxv., aquæ f.ʒiij. At 10:15 skin warmer, no cramps, no vomiting, pulse fuller. At 10:30 vomited six or eight ounces of fluid like the first. At 10:45 a thin, light-colored, sour and acrid-smelling discharge from the bowels; cramp in the left foot. At 12 M. temperature in the mouth, 97 3-5° F. At 1 P. M. vomited eight ounces brownish liquid; bowels moved soon afterward, much as before. At 1:30 felt quite easy, free from all pain, skin warm, face bright, pulse much better, and general condition good. At 2:45 vomited about two ounces yellowish fluid. At 4 drank some tea; rejected the first part and retained the latter; retained also some bread. 5 P.M.—Pulse, skin and general condition natural; feels quite well and hungry; tongue a little furred; temperature 99°. To stay in bed the remainder of the day, but to take no more medicine.

6th July—Feels perfectly well, but is retained at the hospital as a precaution.

7th July—Remained well all day yesterday. Duty.

This, in my judgment, is so remarkable and, so far as I know, is so entirely without precedent as to treatment, that I prefer to let it stand without comment. We cannot legitimately reason upon a single case, when we remember how wonderful are some of the recoveries that follow the unassisted efforts of Nature.

CASE IX.—J. M., in hospital since 14th June with orchitis, which is now nearly relieved, complained, 6th July, 1874, of constant griping pains in the bowels, with four or five stools, small, hard and painful, with straining, in the course of the day. The last occurred at 4 P.M., since when he has had constant griping, intestinal pain. At 9 P.M. gave pulv. ipecac. gr. xxv., shortly preceded by tr. opii *m.* xxv. The abdominal pain ceased in half an hour; he went to sleep about 10 o'clock and slept soundly all night, with no nausea and no movement of the bowels.

7th July—Entirely free from all pain, and feels well.

P.M.—Pains recurred much as yesterday. Gave experimentally twenty-five grains of ipecacuanha, without laudanum or mustard.

8th—The medicine was retained, but the bowels moved several times with some general pain and rectal suffering. The abdominal pain continuing, gave ol. ricini f.℥i., tr. opii *m.* v. Several passages followed, but the suffering was not relieved. Toward evening both pain and diarrhœa continued with some straining. At nine o'clock gave tr. opii *m.* xx., followed by pulv. ipecac. gr. xxv. The pain ceased in half an hour; he fell asleep at ten o'clock and slept well all night, having some perspiration toward morning.

9th.—Entirely free from pain; bowels not moved.

P.M.—Bowels were quiet all day till four o'clock, when and at five o'clock, slight motions, with pain, occurred. A dose of Squibb's diarrhœa mixture given at nine o'clock.

10th.—Slept well, and had no further trouble, except from a slight attack of piles. Duty, 15th July.

The foregoing case is given for what it is worth. Some may chiefly attribute the relief to the laudanum; but, taking it in connection with the series, I think that the ipecacuanha may claim a share. The dose on the night of the 7th, without an adjuvant, clearly shows that twenty-five grains of ipecacuanha is not necessarily an emetic, even when no tolerance has been previously established.

The following case indicates that it may sometimes be used to relieve disagreeable symptoms in the course of a continued fever.

CASE X.—R. B. G. is now well advanced in the first week of an attack of bilious remittent fever.

6th July, 1874.—During the day he has had four or five painful fluid passages from the bowels; tongue is brown and furred; has taken no purgative for a day or two; anticipates, from his present feeling, a restless and sleepless night.

9 P.M.—Gave tr. opii *m.* xxv., followed by pulv. ipecac. gr. xxv. and a sinapism.

7th July, 9 A.M.—Slept well all night, and had one large, soft, blackish operation, without pain, at five o'clock. Feels very comfortable, has no abdominal uneasiness, and tongue is cleaner.

P.M.—Had some abdominal pain, and was therefore ordered twenty-five grains of ipecacuanha at nine o'clock. This was imprudently given without laudanum or mustard, and, being restless and drinking a little citric acid water, he threw up the medicine in a few minutes. The vomiting was of short duration, and he afterward slept soundly.

He was very comfortable the next morning, and had one loose, yellowish passage, about noon, without the intervention of any laxative. This patient passed successfully through a severe attack of fever, but the drug was not employed with him again.

CASE XI.—F. H. W. had four dysenteric discharges—small, painful, bloody, with straining—during the night of 6th July, 1874. He attributes his condition to unusual diet on 4th. Admitted hospital 7th. 9 A.M.—Feels badly, but has had no passage since five o'clock. Temperature normal. As a precaution, and also as an experiment, was put in bed and given twenty-five grains of ipecacuanha, without opium. 4 P.M.—Very comfortable; skin warm and perspiring; some abdominal pain that existed at admission gradually passed away; was a little nauseated about one o'clock, but did not vomit. 9 P.M.—Gave ten grains of ipecacuanha, without an adjuvant.

8th July.—Retained the medicine last night easily; feels well to-day. 9th July, duty.

This man had a severe attack of dysentery in 1863, beginning exactly like this, which led him to report at once for treatment. He is of excellent character, and his assertions may be relied on.

In the following case I have some doubt as to the truth of those statements that rest solely upon the patient's testimony. The man's character is not good, and it is possible that he exaggerated or fabricated the symptoms related. In that event the case merely shows that large quantities of ipecacuanha may be taken without vomiting.

CASE XII.—F. W. claims to have had troublesome diarrhœa for more than a week or ten days, for which he has taken various ineffectual medicines of his own prescribing. Yesterday the pain was more severe, and in the night he had several small passages, marked with blood.

9th July, 1874. Has gone several times to the closet, with ineffectual straining (tenesmus). Has now constant tormenting pains; no fever; tongue a little coated. 9 A.M.—Given tr. opii *m.* xv., pulv. ipecac. gr. xxv.; cautioned to drink no fluid, and to remain strictly at rest. An hour and a half later was found sitting up in bed playing cards. Was required to lie down, when he fell asleep. 12 M.—Vomited a little bitter fluid, and slept afterward. 3:30 P.M.—Went to the closet and passed urine, but nothing from the bowels; did not strain; had no pain; tongue somewhat coated. 6 P.M.—Given tr. opii *m.* xv. and pulv. ipecac. gr. xxv. At seven o'clock a sinapism was applied for nausea, and at eight o'clock he felt entirely easy in every respect.

10th July.—Vomited about eight ounces bitter (?) fluid, of whitish (?) color, about half past eight last night. In the night had a copious fluid passage, after which he felt entirely relieved. Feels quite well this morning; allowed to get up. No further medicine was used in his case, and he was returned to duty 13th July.

In this connection it may be noted that, treating an officer for a complaint in which constipation was an incidental symptom, he was given, experimentally, 9:30 P.M., 10th July, 1874, tr. opii *m.* xx., followed by pulv. ipecac. gr. xxv. This was retained four hours, during which time he slept, and it was then thrown up with about four ounces of reddish fluid, presumably food. There was no other symptom dependent upon the drug.

The following case is too long and uninteresting to reproduce in detail. This is, however, a faithful abstract of its essential features:

CASE XIII.—J. H. has—10th July, 1874—suffered some time with an obstinate and irregular diarrhœa, not dysenteric, apparently due to hepatic torpor with malarial depression. Nitro-muriatic acid, taraxacum, quinia, and other ordinary remedies were ineffectual. The bowels sometimes moved painfully six times in twenty-four hours. Ipecacuanha, in 20–30 grain doses, was given eight times, sometimes with laudanum and mustard and sometimes without, 10th–14th, inclusive. Five of these doses were followed by vomiting, varying in quantity from three ounces to nearly a pint. Returned to duty 18th, but relapsed and taken again under treatment 22d July. Six doses, from twenty to thirty grains each, were given between 22d and 29th inclusive. Emesis occurred only once, to the amount of twelve ounces, during this interval. Ipecac., gr. vj., was given every six hours, without producing nausea, 30th July—2d August inclusive. Eighty grains, in three doses, was given without emesis between 2d and 5th. Substantially no treatment occurred between 5th and 14th August. At this time the tongue was large and flabby, and there were two or three loose, painless passages daily. Took twenty grains ipecacuanha with laudanum and mustard, 15th, without vomiting and the same 16th, when emesis followed. No treatment from 17th to 25th inclusive. On 26th ordered quin. sulph., pulv ipecac., aa. gr. vj., ext. bellad. gr. ¼, four times a day. The man rapidly improved, and by 11th September he was sufficiently well to join his company, under marching orders, for duty.

The preceding was probably the case least suited for ipecacuanha in the series.

Case XIV.—Captain F. is of delicate constitution and is so readily nauseated that he can excite vomiting by a mental effort. He is subject, especially in warm weather, to attacks like the following, which generally last for more than one day. On the morning of 25th July, 1874, which was very hot, attendance at a court martial, in full uniform, for several hours, fatigued him very much. Between twelve and one o'clock he went into the town of Atlanta, and was suddenly seized with nausea and cramp-like pains in the bowels, compelling his immediate return to the post. He felt better after resting, but on sitting down to dinner, and before eating, the pains returned with increased violence, obliging him to lie down. A flannel saturated with laudanum and spirits of camphor was applied, and the suffering subsided in half an hour, when he fell asleep, awaking in two hours in a profuse perspiration. (The room in which he was lying was quite close.) When cooler he sat up, but instantly the pain returned with great violence, accompanied by nausea. At this time I first saw him. He was at once given twenty grains of powdered ipecacuanha in a very little water, with no adjuvant treatment except enjoining absolute abstinence from motion, conversation, food or drink. He was not told the name of the medicine. In seventy minutes he vomited rather less than half a pint of yellowish fluid, with what appeared to be a part of the powder floating upon it. The nausea was of no duration, and the vomiting was at one effort and not severe. He began to feel easier soon after swallowing the powder, and supposed that he had taken an opiate. After vomiting he felt entirely relieved, and two hours later drank some tea. He slept quietly through the night, and went to duty in the morning. A large semi-fluid operation from the bowels (color not noted) occurred at 9 A.M. Captain F. volunteered the remark that no previous attack had yielded so readily.

In the foregoing case there was no known error of diet, but the exciting cause appears to have been fatigue and solar heat, acting upon a delicate subject.

CASE XV.—B. R. (*See case V.*) This man had been returned to duty, from sick with diarrhœa, 9th July, 1874. On careful inquiry it proved that he had had some trouble with his bowels during the entire interval, but not enough to prevent him from attending to duty. He however came again on sick report 27th July, saying that he had been taken sick at 4 A.M. the day before, when he first noticed blood in his stools. Had abdominal pain, with numerous small, painful discharges, with straining (tormina and tenesmus,) throughout the day. Estimates that he had at least twenty stools between reveille and taps. Slept hardly at all last night, by reason of the disease. Thinks that he went to the sink ten times in the night. Went three times between reveille and sick call (5:45 A.M.). Admitted hospital 6:30 A.M. Between that hour and eight o'clock had two stools. All of these, say thirty-five in twenty-eight hours, were small and accompanied by pain and straining. He described them as bloody also. Those passed in hospital were small, of a jelly-like or glairy fluid, uniformly reddish throughout, but not streaked with blood. There was pain in the abdomen, the tongue was covered with a thin white fur, the skin was harsh and dry, the pulse somewhat rapid and wiry and the general appearance one of fever, although the thermometer under the tongue only indicated 97 3-5° F.

8:30 A.M.—Given tr. opii *m.* xv., followed in twenty minutes by pulv. ipecac. gr. xxx. and a sinapism. 2 P.M.—Rested perfectly quietly and easily all the morning, entirely free from pain and from nausea. The bowels moved somewhat copiously but without pain at 1:30 P.M. This discharge was perfectly liquid, and free from the glairy character of the previous ones; it was yellow in color and with it were particles resembling curdled milk that floated on the top. His skin was moist and gently perspiring, and his pulse soft. 3:15 P.M.—Bowels again moved. 3:20.—Ipecac. gr. xxx. without tr. opii and with a sinapism. 4:20.—Vomited about two ounces. 5:45—Bowels moved, and ten minutes later four ounces was thrown from the stomach. 6:30.—Vomited about four ounces, and a stool followed. 8.—Temperature 99°; stools examined and found like the one previously described; has no pain and no special uneasiness; tongue moist and cleaning off; has neither eaten nor drank in the hospital, and is now thirsty but not hungry. 9 P.M.—Tr. opii *m*. xx., followed by ipecac. gr. xx.

28th July.—Vomited in the night at 11 and 2 o'clock, quantity and character undetermined; had two painless passages in the night; bowels moved at 8.30 A.M.—stool tolerably copious, fluid, yellowish, with some of the curdled milk appearance. Temperature 98 3-5°; has no pain of any description, and is comfortable; pulse natural; skin moist and pleasant. 1:20 P.M. Bowels moved, much as before. Bowels moved at 4:30, 5:30, 6:30, P.M.; discharges thin, but more natural in color; there was a little straining, but no pain. 9 P.M.—Tinc. opii *m*. xv., followed by pulv. ipecac. gr. xxv.

29th July.—Bowels moved at 11 P.M. and 4 A.M. without pain; discharges liquid, but natural; no emesis; tongue normal; feels well. Ordered a dose of Squibb's diarrhœa mixture at 8:30, and of Hope's mixture at eleven o'clock.

Evening.—Had four small brownish, fluid passages, approaching the natural color, with very slight pain, during the day. 8 P.M.—Hope's mixture, f.ʒj. 9 P.M.—Ipecac. gr. xxv.

30th July.—Three thin, dark discharges before daylight; vomited at 3 A.M.; bowels moved at eight o'clock; tongue clean and natural. ℞. Acid. sulph. arom., tr. opii c., aa. *m*. xv. aquæ, f.℥ij. every two hours. P.M.—Bowels moved twice, without pain, since dinner.

In this case the bowels gradually acquired their natural tone, chiefly under the use of mineral tonics; the man's general feeling was healthy and comfortable at all times. He was returned to quarters 4th, and to duty 11th, August.

The particulars of the following case are from the notes of Acting Assistant Surgeon, S. S. Beach, who had charge of it, and who kindly supplied me with them.

Case XVI.—R. T., not quite three years old, the daughter of an officer, is reported, 4th August, 1874, to have had very loose bowels for the past ten days, the motions consisting of undigested food and watery secretions. Found her in the evening with great thirst; strong craving for fruits and vegetables, which had been largely indulged; fever, reported to have occurred every day for the past three; pulse 110; lips scarlet; tongue red at the edges and tip, covered with a closely-lying, light-brown coat, from the centre back; has six to nine stools every twenty-four hours—generally two in the night—preceded by some pain, and followed a disposition to strain; character like the first, but with more mucus, no blood. Dr. Beach observes: "Ordinarily I should have given a dose of calomel and Dover's powder, followed by castor oil, laudanum and turpentine; but, learning of the successful use of ipecacuanha in similar cases," he ordered pulv. ipecac., gr. vij., in a paste with water, preceded fifteen minutes by tinc. opii camp. *m*. x. on going to bed. Withheld all drink, except cold tea in small quantities.

5th August.—For half an hour last night the child complained of thirst, then slept till 2 A.M., perspiring freely. Had two stools, half an hour apart, similar to the former ones, except that there was less watery mucus and pain. At seven o'clock had a painless discharge, resembling the ipecacuanha paste. 10 A.M.—Pulse 90; skin cool and moist; lips less red; edges of tongue more pale, coat white and raised; less thirst; general appearance bright and playful. Allowed only cold tea, dry toast and boiled rice. No medicine. 7 P.M.—Had no fever to-day; lips paler; tongue cleaner and paler; drank nothing but tea to-day; continues bright and playful; had two slight evacuations from the bowels, both natural and without pain or straining, since 7 A.M.

This child regained its usual health by simple attention to its diet.

As it resulted, probably some other would have been better practice in the following case; but it is recorded as an illustration of the subject.

Case XVII.—Mrs. . . . ., aged about twenty, the wife of an officer.

Evening, 6th August, 1874.—This lady, who has not heretofore been under my care, tells me that she has suffered a good deal during the summer and has been treated for an affection of the liver. Yesterday and to-day has had much pain in the hepatic region, where there is tenderness on pressure; has had pain under the shoulder blades; and to-day there have been severe recurring pains across the abdomen, about the level of the umbilicus. The bowels are regular, and the appetite, until to-day, has been fair. The tongue is not coated, but is marked by the teeth. Has been feverish to-day, and has drank much water. Directed, on going to bed, to take morph. sulph., gr. 1-6, followed in ten minutes by a sinapism, and, as soon as that begins to be felt, by pulv. ipecac., gr. xx, in a teaspoonful of water.

7th August.—The patient took the medicine as directed, and soon fell asleep. After exactly one hour's sleep, she awoke violently sick, and vomited, with great effort, one quart of thin, yellow, sour (not bitter) liquid. There was much straining, and some blood was thrown up at the close of the effort. It appears that in her case emesis is always violent and painful. There was, however, neither any preliminary nausea nor any after the vomiting ceased. In three-quarters of an hour she was again asleep, and she slept until morning. Awoke feeling very weak and faint, but is much better after breakfast. At 7:30 A.M. had a copious, painless, greyish operation from the bowels. Tongue a little less flabby; no fever; soreness remaining over the liver, but no acute pain as yesterday; the abdominal pain continues in the form of occasional griping, but is less severe than yesterday. ℞.—Potas. bromid. ℨj., aquæ. f.℥j: a teaspoonful occasionally.

P.M.—There is some hepatic soreness, but the abdominal pain has ceased. She has been actively engaged in the house and in the town all day, and considers herself well.

This patient went North on a pleasure trip on the night of 10th, up to which time there was no return of the disorder.

CASE XVIII.—Lieutenant B., æt. 23, very robust and of good general health. For several weeks this officer has experienced occasional abdominal pains in the region of the transverse colon, which have gradually increased in frequency and severity. For the last ten days has felt them daily, and twice in that interval has taken mercurial cathartics under the impression of 'biliousness.' Latterly his bowels have been quite loose and his appetite has been poor.

22d August, 1874.—Became thoroughly chilled while freely perspiring, and had much pain in the abdomen and three passages from the bowels.

23d.—Felt pretty well this morning, but about noon had a severe pain near the umbilicus followed by a painful passage. Had four motions from the bowels in the course of the afternoon, and, besides, went several times to stool ineffectually. All of these attempts (successful and otherwise) were accompanied by tenesmus and by considerable abdominal pain. The dejections were moderate in size, and were of a slimy mucus, uniformly colored with blood, with some bloody-looking serous fluid in addition. His head aches, eyes are a little injected, tongue lightly coated with a thin whitish fur, pulse 84, and temperature 100 2-5° F. Ate a light tea at 7 P.M. 9:20 P.M.—Gave tr. opii *m.* xx., and applied sinapism to epigastrium. 9:35.—Gave powdered ipecac. gr. xx. in water f.ℨij. This was retained without nausea for (as he estimates) a little more than an hour, when, having a tickling sensation in the fauces from a slightly elongated uvula and from some catarrhal secretion from the posterior nares—(he had lain perfectly flat upon his back during this interval, and the titillation was thus more easily excited)—the effort of coughing, or the faucial irritation, induced vomiting, and he threw up six or eight ounces of fluid resembling the tea taken in the evening. No nausea followed, and in fifteen minutes the dose was repeated with the laudanum but without the mustard. He immediately fell asleep and slept (as he estimates) about two hours, during which time he perspired profusely. He then awoke with a sensation in the throat like that felt before (he had again been lying on his back), and he also had some nausea. A small amount of fluid was then thrown up with, as it appeared, the medicine. He again slept and perspired, and about 4 A.M. had another slight attack of vomiting. The whole quantity thrown up did not exceed twelve ounces.

24th.—He had no abdominal or other pain in the night, and he had no inclination to go to stool; his headache disappeared; skin moist; felt well but languid; temperature 98°; pulse 70; bowels flatulent but without desire to go to stool throughout the morning; ate an ordinary and moderate breakfast and dinner; about three o'clock had an almost natural operation, closely followed by another. 5 P.M.—Had a thin passage, preceded and followed by some griping, and accompanied by a little straining. Had no other disagreeable feeling. Took a light tea at seven o'clock. At bed-time took pulv. ipecac. gr. xx., pulv. opii gr. j., in pil. iv. No mustard was used.

25th.—The pills caused no nausea nor vomiting. The skin was moist all night, as it has been since the first dose, but there was no excessive perspiration. Ate his usual breakfast. Between 9 A.M. and noon had three loose, yellow passages, with a slight tendency to strain, the last one near its termination being of a light reddish tinge. At 12:30 P.M. took a teaspoonful of Hope's mixture. There were several loose passages in the afternoon, with some uncomfortable feeling, but no blood. At bedtime took pulv. ipecac. gr. x., opii gr. j., in pills, without mustard.

26th.—Slept well all last night; had no nausea whatever. Had one soft, dark passage this morning, without straining or any disagreeable feeling. Has felt well all day. 27th.—Duty.

This officer, in a conversation upon his sensations and upon the effects of the medicine, asserted that he experienced no nausea that appeared caused by the drug, and that he was confident the vomiting on the night of 23d was primarily due to the irritation of the fauces.

ERRATUM.—On page 663, eleventh line from the bottom, insert—"At 11 took pulv. ipecac. gr. xx, aquæ f.ʒij."

(*Continued in next number of Journal.*)

---

